# Integrative options for the management of anxiety and depression in people with cancer

**DOI:** 10.3389/fonc.2026.1896549

**Published:** 2026-10-05

**Authors:** Linda E. Carlson, Julie M. Deleemans, Chantal Savard, Nisha Marshall, Elisabeth T. Irving, Jamie N. Petersson, Hanna S. W. Conradi, Sasha Mallya

**Affiliations:** 1 Department of Oncology, Cumming School of Medicine, University of Calgary, Calgary, AB, Canada; 2 Department of Psychology, Faculty of Arts, University of Calgary, Calgary, AB, Canada; 3 Department of Psychosocial Oncology, Cancer Care Alberta, Arthur J.E. Child Comprehensive Cancer Centre, Calgary, AB, Canada

**Keywords:** anxiety, cancer, depression, mindbody therapy, mindfulness-based interventions (MBIs), relaxation, taichi, yoga

## Abstract

This review summarizes the definition, diagnosis and prevalence of anxiety and depression symptoms and disorders in people with cancer, followed by an overview of integrative therapies that are guideline-recommended for managing these conditions. Recommended therapies include mindfulness-based interventions, yoga, relaxation, music, reflexology, Taichi/Qigong, acupuncture, lavender aromatherapy and hypnosis. This is followed by a summary of emerging therapies that show preliminary evidence of efficacy, but are not yet included in guideline recommendations, including other forms of aromatherapy, light therapy, biofield therapies, psychedelic-assisted therapy and nature-based interventions. The paper concludes with a summary of common methodological issues and suggestions for future research directions.

## 1 Introduction

### 1.1 Anxiety and depression symptoms

Anxiety and depression are among the most common psychological comorbidities experienced by adults living with and beyond cancer, affecting patients across the disease trajectory. The global prevalence of elevated anxiety and depression symptoms among people with cancer is estimated at 30.6% and 33.2%, respectively (1). While these conditions negatively impact quality of life on their own, anxiety and depressive disorders in cancer are also linked to reduced treatment adherence, greater healthcare use, and poorer survival outcomes, including a significantly higher risk of suicide (2–4). Despite these implications, psychological symptoms among people with cancer remain widely under-recognized and undertreated (5, 6). As such, it is important to first distinguish between common cancer-related symptoms of anxiety and depression and formal anxiety and depressive disorders to guide appropriate care.

Anxiety symptoms across the cancer trajectory often manifest as fear at the time of diagnosis, anxiety around the time of surveillance (i.e., “scanxiety”), concern about prognosis, and fear of cancer recurrence (FCR) (5). Though these anxiety symptoms would not meet diagnostic criteria for a formal anxiety disorder, they can still adversely affect quality of life, treatment adherence, and health care utilization (5).

Depressive symptoms, such as feelings of sadness, loss of pleasure, grief, and thoughts of death can be entirely expected responses to receiving a cancer diagnosis, undergoing treatment and recovery, and facing the very real possibility of death and dying (2, 5, 7). Depressive symptoms, even when they do not meet full diagnostic criteria for a depressive disorder, still negatively affect quality of life, treatment adherence, and possibly survival (7).

Among the factors that contribute to anxiety and depression, FCR and death anxiety are particularly prominent. Up to 40-50% of cancer survivors report moderate-to-severe FCR (8), which can exacerbate anxiety and contribute to the onset or persistence of depression symptoms (9). Death anxiety similarly affects both survivors and patients approaching end of life and has been associated with elevated depressive symptoms (10, 11).

### 1.2 Anxiety and depressive disorders

Anxiety disorders, such as generalized anxiety disorder (GAD) and illness anxiety disorder, by contrast, are distinct disorders that must meet specific diagnostic criteria. GAD is defined by the DSM-5 as excessive, difficult-to-control worry across multiple domains occurring more days than not for at least six months, accompanied by symptoms such as restlessness, fatigue, poor concentration, irritability, muscle tension, and sleep disturbance, and causing clinically significant distress or functional impairment (12, 13). Although there is overlap in symptoms, distinguishing GAD from expected cancer-related anxiety symptoms requires attention to the pervasiveness, controllability, and duration of symptoms: in GAD, worry is persistent and difficult to control across multiple life domains beyond cancer, whereas cancer-related anxiety symptoms tend to be situation-specific and time-limited (12, 14).

Illness anxiety disorder is characterized in the DSM-5 as a preoccupation with having or acquiring a serious illness that is disproportionate to any actual medical findings. In people with cancer, this diagnosis applies when health-related anxiety is clearly excessive relative to the severity of the medical condition and persists beyond six months (12). FCR shares features with illness anxiety disorder including hypervigilance to bodily sensations, intolerance of uncertainty, and reassurance-seeking, but it is a relatively distinct psychological construct not listed in the DSM-5 (15, 16). Both the American Society of Clinical Oncology (ASCO) and NCCN guidelines recommend routine screening with validated instruments such as the GAD-7 to identify patients whose symptoms warrant further evaluation and intervention (2, 17).

Major depressive disorder (MDD) requires the presence of five or more specified symptoms, including depressed mood or anhedonia, persisting for at least two weeks, most of the day, nearly every day, and causing clinically significant functional impairment (12). Diagnosing MDD in people with cancer is often challenging because symptoms often overlap substantially with the physical impacts of cancer and cancer treatment, e.g., fatigue, weight loss, appetite changes, sleep disturbances, and poor concentration (7, 18). Therefore, experts recommend placing greater diagnostic weight on the psychological and cognitive symptoms (i.e., anhedonia, worthlessness, excessive guilt, hopelessness, and suicidal ideation) when assessing people with cancer (12, 18). Both ASCO and NCCN guidelines recommend routine screening with validated instruments such as the PHQ-9 to distinguish patients with clinically significant depression from those experiencing expected depressive symptoms (2, 6).

This distinction between symptoms and diagnosable disorders is important when considering integrative therapies for anxiety and depression in cancer care. Current clinical practice guidelines adopt a stepped-care approach, matching intervention intensity to symptom severity (2). The joint SIO-ASCO Guideline on Integrative Therapies for Anxiety and Depression, for instance, specifically targets *symptoms* of anxiety and depression in its recommendations, rather than formally diagnosed disorders (5, 19). Similarly, the NCCN Survivorship Guidelines recommend integrative therapies for patients with cancer-related “distress”, while recommending referral to mental health professionals and consideration of pharmacotherapy for those meeting diagnostic criteria for anxiety and depressive disorders (6). This reflects the fact that the recommendations are based predominantly on trials measuring symptom change on validated scales (e.g., HADS, PHQ-9, GAD-7) rather than from studies requiring a formal psychiatric diagnosis for inclusion (5). Recognizing where someone falls on this spectrum is therefore essential for selecting appropriate integrative options for management and setting realistic therapeutic expectations.

### 1.3 Prevalence of anxiety and depression

Estimates of anxiety and depression prevalence among people living with cancer (PLWC) vary widely due to differences in assessment methods, diagnostic thresholds, cancer populations, and timing of evaluation. Despite this variability, prevalence rates consistently exceed those observed in the general population (20). Importantly, anxiety and depression are not static experiences; they can fluctuate across the cancer continuum and are influenced by disease characteristics, treatment burden, prognosis, and patient demographics.

Anxiety and depression may arise at any stage of the cancer trajectory, including diagnosis, active treatment, survivorship, and end-of-life care. Although findings regarding vulnerability across this trajectory remain mixed, anxiety tends to be more prominent early in the cancer experience, particularly around diagnosis and treatment uncertainty, whereas depressive symptoms may emerge or intensify over time as physical, emotional, and functional burdens accumulate. One systematic review and meta-analysis of prostate cancer patients demonstrated this fluctuating pattern: pretreatment, on-treatment, and post-treatment prevalence rates for depression were estimated at 17.27%, 14.70%, and 18.44%, respectively, while anxiety prevalence rates were 27.04%, 15.09%, and 18.49% (21). In contrast, other evidence suggests relatively comparable rates of anxiety and depression across stages of treatment, including among patients in active treatment and those receiving end-of-life care (22).

### 1.4 Subgroup prevalence – cancer type, demographics

The prevalence of anxiety and depression also varies substantially across cancer types. Cancers associated with poor prognosis, severe symptom burden, or significant functional impairment are generally linked to higher levels of anxiety and depression. For example, lung, gynecologic, hematologic, and pancreatic cancers are associated with elevated anxiety and depressive symptoms due to their aggressive disease trajectories and limited survival outcomes (23, 24). Similarly, greater overall symptom burden has been identified as a predictor of both depression and anxiety (25). Head-and-neck cancers may contribute to anxiety and depression through visible disfigurement, as well as impairments in speech and sensory functioning (26, 27). In breast cancer, body image concerns, including those experienced following breast reconstruction, as well as sexual dysfunction, have been identified as significant contributors to depression and anxiety (28–30). Certain cancers may also biologically contribute to mood disturbances through neurological and biochemical mechanisms, such as direct neurological involvement in brain tumors (31) or cytokine and hormone dysregulation in pancreatic and lung cancers (22). Treatment-related factors, including chemotherapy and corticosteroid use, have likewise been associated with higher rates of depression (22).

Demographic and psychosocial factors further shape psychological outcomes among PLWC. Women generally report higher rates of anxiety and depression than men, at rates reaching two to three times greater in some cancer types (23). However, findings regarding the relationship between age and anxiety and depression in PLWC are inconsistent, although a study by Noel et al. (32) found that younger adults with cancer are at a higher risk for psychiatric illness and non-fatal self-injury relative to older adults with cancer; Though other studies typically report mixed findings between age and rates of anxiety and depression (23, 33, 34). Other social determinants including limited social support, financial hardship, and racialized minority status are consistently associated with increased anxiety and depression symptoms in PLWC (35–37). Regional variation has also been observed internationally. For example, the highest rates of anxiety and depression among patients with breast tumors have been reported in the Eastern Mediterranean region, and the highest prevalence of depression among digestive cancer populations has been identified in South-East Asia (38). Similarly, rural residence has been identified as a significant predictor of anxiety and depression among breast cancer survivors (25). Taken together, the evidence suggests that anxiety and depression among PLWC are multifactorial and dynamic phenomena shaped by interactions among disease characteristics, treatment experiences, and broader demographic and contextual factors.

## 2 Review methodology

For section 3, guideline-recommended summaries, oncology guidelines from ASCO, SIO, NCCN, MASCC and ESMO were searched for recommendations that included integrative therapies for treating anxiety and depression symptoms in people with cancer. Integrative therapies were broadly defined as any mind-body therapies, natural health products, manipulative therapies, and biofield therapies. Exercise and nutrition were specifically excluded because these topics were the focus on other papers in this invited collection. The therapies covered in Section 4, Emerging Therapies, were chosen among those listed as “inconclusive” in the SIO-ASCO guidelines. This was because the criteria to be mentioned as “inconclusive” in the guidelines included there being some published RCTs on these topics, but not enough research to make specific recommendations. Other emerging therapies outside this list were chosen based on our experience with these therapies and awareness of emerging data. However, a comprehensive or systematic search of therapies was not conducted, so this should be considered a selective group of emerging therapies chosen based on recent evidence and expert opinion.

## 3 Guideline recommended integrative treatment modalities

### 3.1 Mindfulness-based interventions

Mindfulness-Based Interventions are typically defined as programs based on Mindfulness-Based Stress Reduction (MBSR) first developed in the late 1970s by Jon Kabat-Zinn (39) and later popularized, adapted and evaluated across many medical settings (40). They are multi-week group-based meditation programs that combine daily intensive mindfulness meditation practice with mindful movement and education around the stress response, mindful coping and other related topics.

Clinical guidelines from the ASCO and the NCCN recognize mindfulness-based interventions (MBIs) as an essential part of supportive care for distress in adults with cancer. The joint Society for Integrative Oncology (SIO)-ASCO Integrative Oncology Guideline (5) provides a strong endorsement of MBIs for anxiety, indicating that MBIs should be offered to adults with cancer to improve anxiety symptoms during active treatment and in the post-treatment period (quality of evidence: high; strength of recommendation: strong). This recommendation draws from seven systematic reviews, including between 1,094 and 3,476 participants. Effect sizes in the short term were generally in the medium size range. Effect sizes in the medium term (≤6 months) were slightly smaller, and insufficient data were available to determine efficacy beyond this period. The strongest evidence was derived from studies of women with breast cancer, although the recommendation applies broadly across cancer types. Both in-person and virtual formats demonstrated efficacy (5).

The NCCN Distress Management Guideline (v2.2026) (6) also includes mindfulness among recommended psychosocial interventions for managing distress (including fear and worry about the future), although these guidelines do not provide as strong or anxiety-specific an endorsement as the SIO-ASCO recommendations. A recent 2026 meta-analysis of 45 randomized controlled trials (RCTs) (N = 7,395) confirmed that MBIs significantly reduce anxiety (g = -1.06) relative to control conditions and across cancer types, with the strongest effects in breast cancer populations (41).

The SIO-ASCO and NCCN guidelines also recommend mindfulness for managing depressive symptoms. Using the same body of literature cited in the anxiety recommendation, the SIO-ASCO Guidelines (5) once again state that MBIs should be offered to adults with cancer to improve depressive symptoms during and after active treatment (quality of evidence: high; strength of recommendation: strong) (5). Meta-analyses cited in this recommendation demonstrate short-term reductions in depressive symptoms that were mostly in the moderate effect-size range. Fewer trials reported follow-up data, but medium-term effects (≤6 months) ranged from small to large (SMDs 0.26-0.85), with insufficient evidence to determine long-term efficacy beyond 6 months (5).

The NCCN Survivorship Guidelines (v2.2026) (17) includes in-person and virtually delivered MBIs among its recommended approaches for managing distress-related depressive symptoms, including sadness related to loss of usual health, sleep disturbance, appetite changes, difficulty concentrating, and preoccupation with illness-related thoughts. The NCCN Cancer-Related Fatigue Guidelines (v2. 2026) (42) also includes mindfulness among its recommended strategies for managing post-treatment fatigue, a symptom associated with higher rates of depression. Consistent with these recommendations, a recent meta-analysis shows that among adults with cancer, MBIs significantly reduce depressive symptoms relative to active control groups (g = -0.92) (41).

### 3.2 Yoga

Yoga is a mind-body practice that integrates gentle movement, breath work, relaxation, and meditative practice. Although rooted in longstanding contemplative and movement traditions, yoga has increasingly been incorporated into integrative oncology as a supportive care approach for managing the physical and psychological effects of cancer and its treatment (43, 44).

Within the SIO-ASCO guideline, yoga is recommended as an integrative option for symptoms of both anxiety and depression during active treatment and after treatment (5). This recommendation reflects the relatively well-developed yoga literature in breast cancer populations compared with other cancer types, as well as the ability to adapt yoga practices to different levels of physical function, fatigue, and treatment-related limitations.

Evidence for anxiety comes primarily from randomized trials summarized in systematic reviews. In a systematic review and meta-analysis. Gonzalez et al. (45) found that yoga-based interventions were associated with significant reductions in self-reported anxiety symptoms among people with cancer. Across 16 randomized controlled trials including 977 participants, yoga showed a modest-to-moderate effect (*g* = −0.347, 95% CI = −0.473 to −0.221, *p* < 0.001) on anxiety compared with control conditions, with subgroup analyses suggesting that effects were most consistent when yoga was delivered during active treatment (45). Although the authors noted limitations related to study quality and control group differences, the findings from their review support yoga as a promising adjunctive option for anxiety (45).

Similar benefits have been observed for depressive symptoms, with findings from both systematic reviews and individual randomized trials suggesting potential benefits. Gonzalez et al. (45) found significant (*N* = 1,486, *g* = −0.419, 95% CI = −0.558 to −0.281, *p* < 0.001) improvements in depressive symptoms among people with cancer who participated in yoga-based interventions. In an individual randomized trial, Rao et al. (46) compared an integrated yoga program with supportive therapy among women with stage II–III breast cancer undergoing conventional treatment and found greater reductions in self-reported depression scores in the yoga group across surgery, radiotherapy, and chemotherapy. Earlier trial evidence in breast cancer patients receiving radiotherapy also suggested that yoga may improve mood-related outcomes and stress physiology, including cortisol rhythm, further supporting its role as a mind-body supportive care strategy during treatment (45, 46).

Overall, yoga can be described as a guideline-supported complimentary intervention for anxiety and depressive symptoms in adults with cancer, particularly among people with breast cancer (5). Its relevance in oncology likely reflects the combination of gentle movement, breathing practices, relaxation, and attentional training, which may help patients manage distress while also addressing related concerns such as fatigue, sleep disturbance, and reduced physical functioning (44). In practice, yoga should be adapted to the person’s treatment phase, symptom burden, mobility, surgical history, bone health, balance, and overall functional status (44). It should be framed as complementary to evidence-based psychosocial, psychiatric, and pharmacologic care when clinically indicated, rather than as a replacement for these approaches (5, 44).

### 3.3 Relaxation therapies

Relaxation therapies consist of progressive muscle relaxation (PMR), breathing exercises, guided imagery, autogenetic training, biofeedback, and self-hypnosis (47, 48). Many of these interventions aim to reduce physiological arousal through an intentional focus on body awareness, muscle tension and breathing patterns. PMR, for instance, involves intentionally tensing and releasing different muscle groups throughout the body in combination with breathing techniques (49). It has been widely used in a variety of settings due to its ease of learning and limited side effects, additionally, PMR has shown promising effects in reducing negative affect, particularly anxiety (SMD range = -1.32 to -1.09) and depression (SMD = -1.43), in oncology populations (50–52).

Similarly, breathing exercises, such as slow breathing or diaphragmatic breathing, involve intentionally changing the rate, pattern and depth of breath (53, 54). These practices can be advantageous when used in isolation as they are accessible, inexpensive and associated with minimal side effects (53, 54). Additionally, they may improve anxiety and depression in breast cancer-specific populations (47, 53). More broadly, relaxation therapies have been graded as having moderate evidence for reducing for anxiety (C) and strong evidence for reducing depression (A) (47, 53).

Other relaxation therapies emphasize attention and self-awareness. Guided imagery typically involves first relaxing the body using breathing or other techniques described above, then directing ones focus to pleasant imagery, such as a relaxing nature scene or a safe place. Guided Imagery of this nature has been associated with reductions in anxiety symptoms (47, 55). Likewise, autogenic training, the practice of focusing on various physical sensations throughout the body, has shown potential benefit for symptoms of depression and anxiety in cancer populations (47, 56). Biofeedback is similar to autogenic training but uses monitoring devices to facilitate self-regulating one’s physiological activity, such as heart rate. Biofeedback has demonstrated limited, but promising effects in reducing depression and anxiety among cancer patients, although further research is needed to establish its effectiveness (57).

Overall, the various relaxation therapies have demonstrated benefits in both symptoms of depression and anxiety and are recommended in the SIO-ASCO guidelines for patients undergoing active treatment, with weak and moderate recommendations, respectively, while simultaneously offering low risk and low-cost complementary therapies that are safe during a patient’s cancer journey (5, 48).

### 3.4 Music therapy

Music therapy refers to interventions using music delivered by Certified Music Therapists to address a wide variety of therapeutic goals (e.g., cognitive, communicative, emotional, musical, physical, social, and spiritual domains) (58). Music therapy is evidenced to be effective in a wide variety of settings and populations. It involves engagement with music ranging from passive interaction with music (e.g., listening) to more active participation in the music process (e.g., music creation: singing, writing lyrics and playing instruments, and interpreting music through movement) (58, 59). Clinical practice guidelines (i.e., SIO-ASCO) recommend music therapy for the treatment of anxiety and depression in adults with cancer during active treatment (5, 48), though it is noted that the quality of evidence is low and the strength of recommendation is moderate with benefits outweighing harms (5).

There are several advantages to music therapy. In particular, passive interventions such as music listening are relatively easy to implement for patients undergoing cancer treatment. In hospital settings, listening to music can evoke positive emotions and promote relaxation, helping individuals feel calmer, happier, and more at peace, which may reduce anxiety and depression (59). Additionally, music therapy interventions can facilitate emotional expression and improve communication surrounding patients’ experiences. Group-based music therapy interventions may also foster a sense of community and belonging, further contributing to reductions in anxiety and depression (59).

Empirical investigations examining the mechanisms underlying music-based interventions remain limited, particularly in oncology populations. However, a 2025 review examining mechanisms of music therapy for late-life depression provided useful insights into how music therapy may exert its effects, including through improvements in emotion regulation, social engagement, and positive cognitions (60).

Although music therapy has several clear advantages and is recommended in clinical practice guidelines, limitations within the evidence base remain. A recent review of reviews found inconclusive evidence regarding the effects of music therapy on depression and anxiety, largely due to methodological limitations in the included reviews (61). However, the review concluded that music therapy was effective in improving mood and reducing distress more broadly among people with cancer. Overall, current evidence suggests that music therapy is safe, more effective than usual care alone in improving mood and distress and is recommended for treating symptoms of depression and anxiety in people with cancer.

### 3.5 Reflexology

Reflexology involves the application of targeted pressure to specific “reflex points” on the feet or hands that are hypothesized to correspond with internal organs and body systems (62). Reflexology uses focused pressure techniques applied to designated reflex areas (63). Proposed mechanisms of action include modulation of neural pathways, increased blood flow, reduced sympathetic nervous system activity, enhanced parasympathetic responses, endorphin release, and promotion of relaxation and homeostasis (64). Reflexology has increasingly been integrated into supportive care settings as a non-pharmacological approach intended to support patient well-being (62, 64).

In the context of cancer, reflexology has been investigated as a complementary intervention for reducing anxiety and depression among people with cancer. Recent clinical guidelines developed jointly by SIO and ASCO state that reflexology may be offered to people with cancer to improve anxiety and depression symptoms during active treatment, although the quality of evidence was considered low and the strength of recommendation weak (5). Similar recommendations have been extended to post-treatment and palliative settings (19), although more research is needed particularly for patients facing a terminal cancer diagnosis.

Evidence for the use of reflexology to address anxiety during active cancer treatment is derived primarily from RCTs conducted across breast, gynecologic, gastrointestinal, and mixed cancer populations (5). Reflexology for depression has mainly been conducted in studies with breast and gynecologic cancers (5). Studies involving gynecologic and mixed cancer populations have reported small-to-moderate reductions in anxiety scores. Additionally, a systematic review by Klaus et al. (65) which included 26 studies and 2,465 participants, concluded that reflexology appeared more beneficial than passive control conditions for outcomes including anxiety, depression, pain, fatigue, and quality of life, though methodological variability limited interpretation. Likewise, Calcagni et al. (66) reported mixed findings across reflexology studies in oncology populations, with overall high risk of bias.

Importantly, reflexology appears to have a favorable safety profile, with no significant adverse events reported across oncology studies (5, 65). Hence, reflexology may represent an accessible, low-risk, supportive care option for patients with cancer experiencing anxiety and/or depression. Nevertheless, caution is warranted as current evidence does not support reflexology as a primary intervention for cancer-related anxiety, and modalities with a stronger evidence base remain preferred first-line integrative approaches.

### 3.6 Taichi/Qigong

Tai chi and qigong are mind-body movement practices rooted in traditional Chinese health traditions. While tai chi has origins in martial arts and is characterized by slow, flowing movement sequences, qigong more broadly emphasizes coordinated movement, breathing, posture, relaxation, and focused attention. In cancer care, these practices have been adapted into accessible programs that can be delivered in standing or seated formats, making them relevant for people managing treatment-related limitations, deconditioning, or reduced physical function (5).

Within oncology, tai chi/qigong has largely been studied in post-treatment survivorship. This is reflected in the SIO-ASCO guideline, which recommends tai chi/qigong for anxiety and depressive symptoms after cancer treatment, while noting that evidence during active treatment remains insufficient to support a recommendation (5). Although most studies have been conducted in breast cancer survivors, limiting generalizability to other cancer populations, this evidence remains highly relevant to survivorship care. These studies evaluate anxiety and depressive symptoms alongside common post-treatment concerns such as fatigue, sleep disturbance, reduced physical function, and quality of life.

Among psychological outcomes, anxiety has shown the clearest signal of benefit. In a systematic review of 15 randomized controlled trials involving 1,156 women with breast cancer, Li et al. (67) reported that Tai Chi Chuan significantly reduced anxiety compared with control conditions (WMD = −4.25; 95% CI: −5.88 to −2.63; I² = 0%). The review also found improvements in fatigue, and quality of life although no between-group effects were observed for depression, sleep quality, cognitive function, or inflammatory markers. These findings have been supported by more recent evidence. A systematic review and meta-regression of tai chi/qigong interventions in adults with cancer similarly reported small-to-moderate effects (standardized mean effect size = 0.29; 95% CI, 0.18 to 0.40) on anxiety symptoms, suggesting that these practices may be useful for survivors experiencing persistent worry, physiological arousal, or emotional distress in the post-treatment period (68). Taken together, these findings suggest that tai chi/qigong may be particularly relevant for survivors experiencing persistent worry, physiological arousal, or emotional distress in the post-treatment period (69).

Evidence for depressive symptoms is encouraging but less definitive. Chen et al. (70) conducted a systematic review and meta-analysis of 16 randomized controlled trials involving 1,247 participants and found that Tai Chi and Baduanjin, a structured form of qigong, significantly reduced depressive symptoms (SMD = −1.44; 95% CI: −2.46 to −0.41; P = .006; I² = 88%). The same review also reported improvements in fatigue, sleep quality, cognitive function, shoulder function, and quality of life. Rather than suggesting a mood-specific effect alone, these findings point to the possibility that depressive symptoms may improve as part of a broader pattern of gains in rest, energy, physical functioning, and perceived well-being. This interpretation is supported by meta-regression evidence suggesting that antidepressant effects may be stronger when tai chi/qigong is accompanied by improvements in function-related outcomes (68). However, confidence in these findings remains limited by small sample sizes, heterogeneity across intervention protocols, variability in comparators, and concerns about overall study quality (67, 69, 70).

More recent pragmatic trial evidence also supports tai chi/qigong as a survivorship-focused intervention for mood and distress. The MATCH study found that both mindfulness-based cancer recovery and tai chi/qigong improved mood in distressed cancer survivors, regardless of whether participants selected their preferred program or were randomized (71). These findings are particularly relevant to survivorship care, as patient preference, acceptability, and long-term engagement may influence adherence to self-management strategies and support the sustained use of these practices beyond the structured intervention period.

Overall, the evidence is consistent with SIO-ASCO’s post-treatment recommendation and suggests that tai chi/qigong may be a feasible and acceptable a complementary integrative therapy for anxiety and depressive symptoms in the post-treatment setting (5). Its clinical appeal lies in the combination of gentle movement, breath-focused relaxation, and attentional training, which may be especially suitable for patients who prefer movement-based approaches or who may not tolerate more strenuous exercise. At the same time, the current evidence base remains strongest in breast cancer survivorship and should be interpreted with attention to study quality and intervention variability. As with other integrative therapies, tai chi/qigong should be offered as a complement to evidence-based psychosocial, psychiatric, and pharmacologic care when clinically indicated.

### 3.7 Acupuncture

Acupuncture is a component of Traditional Chinese Medicine with origins dating back as early as 100 BCE (72). Used as a method to treat disease and relieve pain, traditional practices suggest that acupuncture can modify the flow of energy through the body, called qi, by inserting thin needles into specific anatomical points, referred to as acupoints, to remove energy blocks and restore balance (72, 73). Within contemporary research, acupuncture is thought to stimulate sensory pathways and contribute to changes in both physiological and psychological processes (74–76).

Electroacupuncture, a variation of traditional acupuncture, involves the application of a low-intensity electrical current (typically ranging from 2 to 100 Hz) through electrodes attached to the inserted needles (73). Both acupuncture and electroacupuncture have been associated with reductions in anxiety, stress, mood disturbances and depressive symptomatology in some clinical populations (47, 77, 78).

Overall, research has shown that acupuncture and electroacupuncture may be associated with reductions in anxiety, particularly in oncology patients; however, few studies have directly established a causal relationship (19, 79–81). More recently, the neurobiological mechanisms have been explored. In rodent models, electroacupuncture was associated with reduced anxiety-like behavior and modifications in brain protein expression in the amygdala and prefrontal cortex (82). In human studies, fMRI evidence indicates reduced regional homogeneity in the amygdala, hippocampus, and areas of the cingulate cortex following electroacupuncture, which may be associated with reductions in anxiety symptomatology (82).

In depression, both acupuncture and acupressure, the application of physical pressure to stimulate acupoints, have been associated with reductions in cancer-related depression (81, 83). However, the overall quality and consistency of evidence remains limited, warranting careful interpretation. Explorations into the neurobiological mechanisms suggest that electroacupuncture may contribute to reductions in inflammatory responses and contribute to the regulation of neuroendocrine systems (84).

Overall, the evidence for acupuncture in the management of anxiety suggests potential benefit both during and after cancer treatment, although the evidence remains limited and few studies have established a causal relationship. Accordingly, the SIO-ASCO guideline provides a weak recommendation for acupuncture for post-treatment anxiety, particularly among women with breast cancer (5).

### 3.8 Hypnosis

Hypnosis has previously been defined as a “state of consciousness involving focused attention and reduced peripheral awareness characterized by an enhanced capacity for response to suggestion” (85). Hypnotherapy is typically administered by a trained clinician (e.g. hypnotherapist, physician or nurse trained in hypnotherapy), though evidence suggests self-hypnosis may also provide an effective strategy in mitigating anxiety associated with cancer (86, 87). This has helped enhance accessibility to hypnotherapy for anxiety, via mobile applications that allow patients to access short self-guided hypnotherapy sessions on demand, wherever they are (88). During hypnosis, the patient is supported in reaching a relaxed state through imagery, suggestions for relaxation and calmness, followed by symptom-specific suggestions, including anxiety reduction (89).

Hypnosis is shown to reduce cancer-related symptoms such as pain, distress, fatigue, and anxiety (77, 90, 91). For instance, in a preference-based nonrandomized trial assessing the short-term effects of three interventions (yoga, CBT, and self-hypnosis) on psychosocial outcomes in people with cancer, self-hypnosis was superior for improving several symptoms, including anxiety and depression (87). Similarly, work by Grégoire et al., found that a group-hypnosis intervention for patients with breast cancer led to reduced anxiety and depression, with effects sustained at up to 9 months post-intervention (92). Importantly, in a meta-analysis of 20 studies using hypnosis to address anxiety in people with cancer, hypnotherapy was shown to have both immediate and sustained effects on reducing anxiety; larger mean effect sizes were found especially among pediatric and blood cancer patients, studies examining procedure-related anxiety, in mixed-gender samples, and when hypnosis was delivered by a therapist rather than self-administered (91).

Evidence supports the use of hypnosis as a complementary, supportive therapy for reducing anxiety and distress among people with cancer. Moreover, studies demonstrate low risk with reasonably high effectiveness. Hence, the SIO/ASCO guidelines state that hypnosis may be offered to people with cancer to improve anxiety symptoms during cancer-related diagnostic and treatment procedures with the quality of evidence set at intermediate, with benefits outweighing harms, and the strength of recommendation being moderate (5). However, acceptance and adoption of hypnosis within standard Western medical oncology care have been slow, prompting the need for larger studies with diverse patient cohorts to support evidence for implementation into cancer care (93, 94).

## 4 Emerging therapies

### 4.1 Aromatherapy

Clinical aromatherapy involves the use of essential oils, often administered through massage or inhalation, for therapeutic purposes. A variety of essential oils (e.g., lavender, lemon) and combinations are used in the practice, each with unique psychological and physiological effects that may vary with their bioactive properties (93, 94). Aromatherapy is increasingly used as an integrative approach aimed at managing psychological symptoms such as anxiety and depression (93–95), although the strength of evidence varies by symptom, treatment phase, and essential oil used. Emerging evidence suggests possible benefits in the use of aromatherapy in managing anxiety in oncology practice, while its potential to alleviate depression remains unclear. Notably, the 2023 SIO-ASCO guideline provides a weak recommendation based on low-quality evidence for the use of lavender essential oil inhalation for anxiety during cancer-related diagnostic and treatment procedures, since the benefits outweigh the harms (5). This recommendation is derived from evidence comprising various cancer types as well as breast cancer-specific populations. The evidence base is still growing, and more recommendations may arise with further research.

Interest in aromatherapy for managing cancer-related anxiety and depression has grown alongside its broader use for mood disorders; however, evidence regarding its effectiveness in oncology settings remains mixed. Recent reviews and meta-analyses report reductions in anxiety with effect sizes ranging from -0.43 to -0.97 (96–98) and, to a lesser extent, depression (98), while others find no significant anxiolytic (99) or antidepressant effects (97). These inconsistencies likely reflect substantial heterogeneity across studies, including variations in essential oil composition, delivery parameters, cancer contexts, and targeted outcomes, which limits the development of clear clinical recommendations.

Methodological limitations further contribute to uncertainty in the evidence base. Few large, rigorous, high-quality trials using validated measures and transparent reporting currently exist (94, 97), limiting reproducibility and integration into evidence-based clinical guidelines. To address these issues, future research should prioritize well-designed randomized controlled trials and transparent reporting practices. The adoption of guidelines, including Consolidated Standards of Reporting Trials (CONSORT) and the Transparent Reporting for Essential oil and Aroma Therapeutic Studies (TREATS) checklist (94, 100), is recommended to improve study quality and comparability.

Despite these limitations, aromatherapy appears to have potential as an adjunctive option for cancer-related anxiety. A recent review by Ahn & Kim (96) suggests that combinations of oils may be more effective than single oils for anxiety. Massage-based delivery is increasingly identified as the preferred technique for treating anxiety in cancer populations (96–98). In contrast, the evidence for depression remains inconclusive. Further rigorous, well-designed studies are needed to clarify optimal intervention characteristics and strengthen the evidence base required to expand aromatherapy recommendations in oncological practice.

### 4.2 Light therapy

Light therapy typically involves exposure to bright white light (BWL) in the morning for 30–40 minutes upon waking over a 3–4 week period. A portable device similar to that used to treat seasonal affective disorder is typically used, which provides up to 10,000 lux of full-spectrum white light. While BWL therapy is not included in any guidelines for treating anxiety and depression, it is mentioned as a potential treatment for cancer-related fatigue in the NCCN fatigue guideline (42).

There have been meta-analyses conducted on BWL which included symptoms of anxiety and depression, although they are typically secondary to fatigue and sleep outcomes. A 2022 meta-analysis of 13 RCTs (551 patients) reported BWL was associated with a statistically significant but small improvement in depression symptoms (SMD = −0.26, P = 0.03), alongside significant improvements in fatigue and sleep difficulty (101). However, a separate 2023 meta-analysis of 12 RCTs (691 patients) found that BWL therapy did not significantly improve depression or quality of life, though it did reduce fatigue and insomnia (102). This discrepancy likely reflects differences in included studies and analytic approaches. An interesting secondary analysis of a trial of BWL versus cognitive therapy for 59 breast cancer survivors showed that women benefited more on depression symptoms when they had no prior history of major depressive disorder, higher depression scores at baseline, and when they received BLT during spring or summer (103).

Anxiety was rarely included as an outcome measure in these trials, with only one recent study of 74 hospitalized patients being treated for acute leukemia showing some benefit on anxiety after two weeks of administration (104).

Nonetheless, while this literature is maturing, like many integrative therapies there is little risk associated with BWL therapy. A 2025 systematic review concluded that BLT is safe and well-tolerated in oncology patients, with minimal adverse effects reported across studies (105).

### 4.3 Biofield therapy

The “biofield refers to a non-physical life energy which is inherent in living systems, reflecting physical, mental, emotional, and spiritual wellbeing (106). Biofield energetic therapies, such as healing touch, reiki, and qigong refer to “non-invasive therapies in which the practitioner explicitly works with a client’s biofield (interacting fields of energy and information that surround living systems) to stimulate healing responses in patients” (107). Biofield therapies have been incorporated into the healing traditions of many cultures for centuries (106), yet due to the “mechanism of action” being challenging to quantify by Western biomedical science at this time, these supportive care strategies have often been considered taboo by Western medical culture. Nevertheless, research on the use of biofield therapies in oncology care has increased, as people with cancer report using biofield therapies as part of their care, and recent studies have yielded promising results to clarify potential mechanisms of action (108).

As of 2023, the SIO-ASCO guideline on integrative oncology care for anxiety and depression in adults with cancer currently notes that there is insufficient evidence to recommend for or against the use of biofield therapies for addressing anxiety or depression among people with cancer (5). Issues with the current evidence base primarily stem from methodological weaknesses, such as small sample sizes. However, studies of biofield therapy interventions for people with cancer have demonstrated potential to address treatment-related symptoms such as pain, fatigue, QOL (109, 110), anxiety (111, 112), and depression (113). Moreover, in a systematic review of 14 randomized placebo-controlled trials, Zadro and Stapleton (114) found that Reiki demonstrated a greater therapeutic effect over placebo for clinically relevant levels of depression and anxiety, with the level of evidence ranging from a grade of moderate to high, though this was not specific to oncology populations.

Importantly, as biofield therapies appear to have a favorable safety profile, with reportedly minimal risk of side effects, further research is warranted.

### 4.4 Psychedelic-assisted therapy

Psychedelic-assisted cancer therapy (PACT) has emerged as a novel approach for addressing anxiety and depression among PLWC. These interventions involve administration of a controlled dose of a psychedelic compound (e.g. psilocybin, lysergic acid diethylamide (LSD), dimethyltryptamine (DMT), 3,4-methylenedioxymethamphetamine (MDMA), ketamine) within a structured therapeutic framework involving preparation, dosing, and integrative supportive therapy.

To date, the strongest evidence for PACT has centered on psilocybin- and ketamine-assisted therapies, whereas evidence for other psychedelic compounds, including MDMA and LSD, remains more preliminary due to a smaller number of studies and less rigorous methodologies. A recent systematic review and meta-analysis reported that both psilocybin- and ketamine- assisted therapies were associated with significant reductions in anxiety and depressive symptoms in PLWC, with ketamine demonstrating rapid and large treatment effects (Hedges’ g = −1.37, 95% CI: −2.66 to −0.08) and psilocybin showing similarly large, though somewhat more variable, effects (Hedges’ g = −3.13, 95% CI: −10.04 to 3.77) (115).

Two of the most influential studies included in this review were randomized controlled trials conducted by Griffiths et al. (116) and Ross et al. (117). Griffiths et al. (116) found that, compared with a very low-dose placebo-like condition, high-dose psilocybin produced large and sustained reductions in depression, anxiety, and death anxiety, alongside improvements in quality of life, life meaning, and optimism among people living with life-threatening cancer. Similarly, Ross et al. (117) reported that a single moderate dose of psilocybin administered with psychotherapy, compared with niacin plus psychotherapy, resulted in rapid and enduring reductions in anxiety, depression, demoralization, and hopelessness, as well as improvements in spiritual well-being, and quality of life in people living with life-threatening cancer. A long-term follow-up of the Ross et al. (117) trial found that 60-80% of participants who received the psilocybin continued to demonstrate clinically significant antidepressant or anxiolytic responses 4.5 years after treatment, suggesting that the benefits of psilocybin-based interventions may extend well beyond short-term symptom reduction (118). Overall, emerging evidence suggests that PACT may offer clinically meaningful improvements in cancer-related anxiety and depression in people living with life-threatening cancer; however, current research remains characterized by substantial heterogeneity in study design, dosing protocols, psychotherapeutic approaches, and outcome measures, limiting definitive conclusions regarding efficacy.

Qualitative findings suggest that PLWC view PACT with cautious optimism, particularly when conventional approaches have inadequately addressed persistent anxiety and depression (115). At the same time, concerns related to safety, stigma, accessibility, and the legal status of psychedelic compounds remain prominent, underscoring the importance of careful implementation and patient-centered models of care (115). Existing evidence regarding safety from controlled clinical trials is generally encouraging. Across three randomized controlled trials of psilocybin-based PACT, no serious physiological or psychological adverse events were reported (116, 117, 119). Although treatment sessions can involve intense emotional or perceptual experiences, these effects are typically manageable with appropriate therapeutic support and clinical supervision (117). Nevertheless, broader safety and implementation considerations remain critical, including careful patient screening, trained facilitators, ethical oversight, and the development of appropriate healthcare infrastructure to support safe and effective clinical use in oncology populations.

### 4.5 Nature-based interventions

Nature-based interventions (NBIs) are an upcoming integrative approach for managing psychosocial symptoms for PLWC. These interventions harness the therapeutic effects of natural environments or elements by instigating behavior change or altering environments to promote health and well-being (120, 121). NBI approaches can include solo or group horticultural therapy, exposure to nature sounds, and physical activity in natural settings; this diversity enables the selection of interventions best suited to target specific outcomes, such as cancer-related anxiety or depression.

Outside of oncology, nature-based activities and prescriptions are gaining traction for treating anxiety and depression. Systematic reviews and meta-analyses suggest that NBIs may reduce these symptoms across diverse populations (122–125). However, much of the current evidence is limited by low methodological quality and poor reporting, which constrains its ability to inform clinical decision-making (123).

In oncology contexts, the evidence base remains comparatively limited but is expanding. Recent reviews indicate that NBIs may help reduce anxiety and depression symptoms in PLWC and survivors (120, 126). Individual, preliminary studies report the beneficial effects of NBIs on anxiety (127–133) and depression (127, 129–131, 133, 134) following a variety of interventions. These NBIs include more passive, incidental exposures (e.g., natural landscape views, nature imagery, virtual reality nature experiences) and more immersive, international multi-modal programs (e.g., nature-focused MBSR programs, forest therapy, wilderness programs, mindfulness-based nature treks, and integrative programs combining yoga, forest exposure, and horticultural therapy). Collectively, these findings suggest that NBIs hold promise as strategies for managing cancer-related psychological symptoms, although the homogeneity of interventions and study designs limits comparability.

Overall, nature exposure is an innovative therapy with the potential to complement conventional oncological approaches, but high-quality intervention research is critical for translating emerging evidence into clinical practice guidelines and facilitating the integration of NBIs into diverse healthcare systems.

### 4.6 Methodological issues and future directions

A number of methodological issues are common across a range of integrative therapies. These include: 1) small sample sizes resulting in underpowered studies with the inability to detect all but large or very large effects; 2) lack of specificity in intervention descriptions and therapist training, precluding replicability; 3) lack of standardization across therapies in a category, harming generalizability; 4) lack of diversity in study samples (across cancer types, stage of disease, sex/gender, race, socioeconomic status) - sometimes known as the “wealthy White Western women” problem; 5) lack of randomized controlled efficacy trials, which are required for inclusion in practice guidelines. Another issue inherent in investigation of therapies in which masking is often impossible is the impact of participants knowing which treatment they are assigned to in RCTs. Researchers in this area need to be aware of the effects of expectancy on outcomes and should measure both expectations for improvement across conditions before intervention participation and measure perceived treatment credibility as well.

Many integrative therapies should be considered complex interventions with multiple interacting components, making mechanistic research attempting to pinpoint the active ingredients contributing to intervention success important (123, 135, 136). These types of mechanistic studies looking at mediators of change are also lacking in the field to date. Studies also rarely systematically report adverse events related to these therapies, rendering assessment of potential harms difficult. Review studies and meta-analyses in these areas should also more thoroughly assess risk of bias and ensure that conclusions are interpreted in light of the quality of the available evidence (61). Overall, studies in these areas often demonstrate methodological weaknesses, inconsistent findings, generally small effect sizes, and modest, inconsistent effects.

Future oncology research should build towards rigorous efficacy trials alongside detailed and transparent reporting of intervention design, contextual factors, and environmental characteristics. The use of established frameworks and reporting guidelines, such as the Medical Research Council (MRC) complex intervention framework (135) and the TIDieR checklist (137), may assist researchers in meeting these goals. Finally, implementation research using pragmatic effectiveness/implementation designs is crucial to expand access for therapies that are already included in guideline recommendations for specific populations. This may include developing and testing remote and online delivery models; work which is already well underway in this space.

## 5 Conclusion

This paper summarizes the range of integrative therapies recommended and emerging with potential to treat symptoms of anxiety and depression in people with cancer. While many therapies are already included in clinical practice guidelines, evidence across more diverse patient samples is still lacking. Meanwhile, implementation strategies for recommended therapies need to be developed, tested and put into practice to ensure a wide range of people with cancer, many of whom suffer from debilitating anxiety and depression, can access these beneficial modalities.
